# Mechanotransducive Biomimetic Systems for Chondrogenic Differentiation In Vitro

**DOI:** 10.3390/ijms22189690

**Published:** 2021-09-07

**Authors:** Ilona Uzieliene, Daiva Bironaite, Paulius Bernotas, Arkadij Sobolev, Eiva Bernotiene

**Affiliations:** 1 State Research Institute Centre for Innovative Medicine, Department of Regenerative Medicine, LT-08406 Vilnius, Lithuania; ilona.uzieliene@imcentras.lt (I.U.); daiva.bironaite@imcentras.lt (D.B.); bernotaspaul@gmail.com (P.B.); 2 Latvian Institute of Organic Synthesis, 21 Aizkraukles Str., LV-1006 Riga, Latvia; arkady@osi.lv

**Keywords:** mechanical load, scaffolds, hydrogels, cartilage, chondrogenic differentiation, mesenchymal stem cells, osteoarthritis

## Abstract

Osteoarthritis (OA) is a long-term chronic joint disease characterized by the deterioration of bones and cartilage, which results in rubbing of bones which causes joint stiffness, pain, and restriction of movement. Tissue engineering strategies for repairing damaged and diseased cartilage tissue have been widely studied with various types of stem cells, chondrocytes, and extracellular matrices being on the lead of new discoveries. The application of natural or synthetic compound-based scaffolds for the improvement of chondrogenic differentiation efficiency and cartilage tissue engineering is of great interest in regenerative medicine. However, the properties of such constructs under conditions of mechanical load, which is one of the most important factors for the successful cartilage regeneration and functioning in vivo is poorly understood. In this review, we have primarily focused on natural compounds, particularly extracellular matrix macromolecule-based scaffolds and their combinations for the chondrogenic differentiation of stem cells and chondrocytes. We also discuss different mechanical forces and compression models that are used for In Vitro studies to improve chondrogenic differentiation. Summary of provided mechanical stimulation models In Vitro reviews the current state of the cartilage tissue regeneration technologies and to the potential for more efficient application of cell- and scaffold-based technologies for osteoarthritis or other cartilage disorders.

## 1. Introduction

Human articular cartilage is a dense, avascular, load-bearing tissue, which is highly susceptible to degenerative diseases such as osteoarthritis (OA). OA has become a global problem not only to senior overweight people with metabolic disorders but also to a physically active younger population and joint injuries [1,2]. Major pharmacological therapeutics for OA are limited to disease-modifying OA drugs (DMOADs), which are insufficiently effective so far to stop OA progression, highlighting the need of more specific and molecular mechanism-regulating treatment [3]. Stem cell therapies have been suggested as one of potential methods for OA treatment; however, direct injections of stem cells into damaged joint do not promote cartilage repair, since transplanted cell are quickly released from the injured joint due to mechanical compression [4]. Therefore, methods of stem cell encapsulation or embedding into damaged cartilage with effective long-term regenerative properties are intensively studied.

The chondrocytes in cartilage are embedded in the dense extracellular network/matrix (ECM) which play a central role in cartilage functioning and its mechanical stability [5]. ECM of hyaline cartilage is composed mainly of collagen type II, peptidoglycans, and glycosaminoglycans (GAGs); therefore, the most models for the cartilage tissue engineering in vitro are ECM components-based hydrogel systems [6]. Biomimetic systems combining various types of stem cells or chondrocytes with natural ECM components, such as collagens and GAGs, have attracted huge scientific attention. Collagen type I-based scaffolds [7] as well as sulfated GAGs, such as chondroitin-4 and -6 sulfates (CS), heparin, keratin sulfate (KS), and non-sulfated GAGs represented by hyaluronic acid (HA) [8] and their combinations [9] are the most popular biomimetic matrices for cartilage tissue engineering and regenerative approaches. Beside the ECM-based components, chitosan, alginate, and their composites can be also used as biomimetic scaffold for cartilage regeneration [10,11].

Today, hundreds of different synthetic, polymer-based scaffolds—such as poly-lactic acid (PLA), poly-glycolic acid (PGA)—and other, or multilayer polymer scaffolds combined with cartilage ECM-related matrix components have been proposed as an efficient matrices for the cartilage regeneration studies [12,13,14]. The natural and synthetic polymers with their physicochemical properties including biocompatibility, biodegradability, morphology, mechanical strength, pore size, and porosity pattern best mimicking the natural cartilage environment in vivo are still leading [15]. Various types of natural or synthetic scaffolds have been tested as cartilage models *in vivo*; however, part of newly tested scaffolds failed to pass mechanical-load stability tests. Many mechanical compression systems have been developed to test the strength and suitability of newly synthesized scaffolds In Vitro for their further efficient therapeutic OA approaches in vivo [16,17]. Therefore, there is an urgent need in modern tissue engineering technologies comprising special crosslinking ways of natural components in order to create mechanically-stable, biocompatible, and biodegradable constructs, carrying cells or drugs suitable for the cartilage regeneration.

The aim of this study is to review the biomimetic single component and mixed ECM-based scaffolds/hydrogels for cartilage tissue engineering In Vitro with particular focus on their mechanotransducive properties.

## 2. Articular Cartilage Damage and the Development of OA

**Figure 1:**
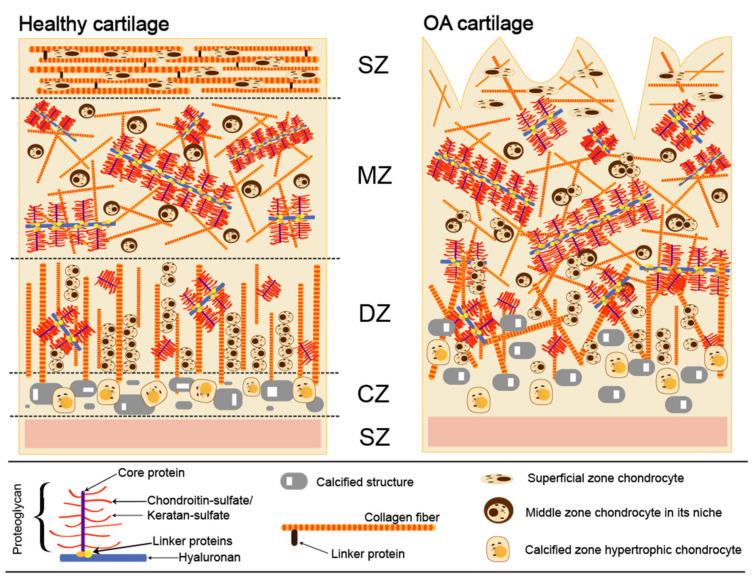
Extracellular matrix in normal and osteoarthritic cartilage. SZ (top)—superficial zone; MZ—middle zone; DZ—deep zone; CZ—calcified zone; SZ (bottom)—subchondral zone.

Cartilage provides a smooth, gliding surface for joint motion and acts as a cushion between bones. Articular cartilage is composed of a dense extracellular matrix (ECM) with a sparse distribution of chondrocytes. There are four different zones in cartilage anatomical section entitled as superficial, middle, deep, calcified, and subchondral (Figure 1) [18]. Although chondrocytes do not directly contribute to the mechanical properties of cartilage, they can sense and respond to various mechanical stimuli within their individual microenvironments. The ECM is principally composed of water (65–80%), collagen (primarily, type II and IX), and proteoglycans, along with other non-collagenous proteins and glycoproteins [19]. Collagen type II is the major type (90% to 95%) of articular cartilage and intertwined with proteoglycan aggregates forms fibrils and fibers. Aggrecan, the largest in size and the most abundant by weight proteoglycan, comprises CS and KS and interacts with HA, another important component lubricating and maintaining mechanical properties of a joint [20]. Each aggrecan contains around 100 CS chains, which are typically ∼20 kDa each and fewer KS chains (up to 60) that are usually of smaller size (5–15 kDa). Aggrecan occupies the inter-fibrillary ECM and provides cartilage with osmotic properties, which are critical for resisting compressive loads. During movement, the water content in healthy cartilage is finely balanced: compressive force drives water out, while hydrostatic and osmotic pressure drives in [21]. Compressive force is maintained by the collagen fibers, while osmotic pressure—by the proteoglycans. However, in OA the collagen matrix becomes disorganized and proteoglycan content within cartilage decreases (Figure 1).

Therefore, therapeutic methods applying stem cells have been considered as one of the potential OA treatments. Different biomimetic scaffolds have been also proposed for cartilage regeneration; however, the majority of such constructs cannot endure mechanical compression as they lack mechanical stability and strength to firmly hold encapsulated cells, which is crucial for chondrocyte homeostasis *in vivo*.

## 3. Tissue Engineering Technologies for Cartilage Regeneration

In order to mimic natural cartilage environment, which is crucial for chondrocyte homeostasis in vivo, chondrogenesis models in vitro should be accordingly optimized. These models contain 3D environment (biomimetic scaffolds) and various types of cells, including mesenchymal stem cells (MSCs), induced pluripotent stem cells (iPSCs) or autologous chondrocytes which are subjected to growth factors and other chondrogenic differentiation promoting compounds, hypoxic conditions, and mechanical stimulus.

MSCs are mostly used stem cells for chondrogenic differentiation studies, due to their easy obtainment and high ability to differentiate into chondrogenic lineage [22,23,24]. These cells are isolated from different tissues, including bone marrow, adipose tissue, umbilical cord, menstrual blood, etc. [25,26,27]. Still, bone marrow MSCs (BMMSCs) are considered as classical cells for cartilage tissue regeneration, as they originate from the closest anatomical location to cartilage tissue, and have a great potential to differentiate into chondrocytes [28]. Autologous chondrocytes are often used in cell therapies as mature cells that could efficiently synthesize cartilage specific proteins however, it is difficult to obtain those cells due to the lack of healthy cartilage.

In addition to MSCs and chondrocytes, pluripotent stem cells also seem to have great potential to repair damaged articular cartilage [29]. Embryonic stem cells seeded on elastic polydimethylsiloxane scaffolds and subjected to mechanical loading resulted in robust induction of chondrogenic differentiation [30]. Embryonic stem cell application-related ethical issues can be avoided by replacement with iPSCs that were also shown to be effective in chondrogenic ECM production [31]. However, the difficulties in directing pluripotent stem cells towards specific mature lineages and iPSCs genomic instability often complicates their use in different applications [32]. Therefore, human MSCs and chondrocytes remain one of the most favorable options for chondrogenesis studies In Vitro.

### 3.1. Chondrogenic Differentiation Protocols

Although cell pellet cultures remain to be a classic chondrogenic differentiation method, chondrogenic differentiation is induced in several different ways [33]. Cell-sheet method by using different biodegradable surfaces, including poly-N-isopropylacrylamide [34], is also getting increasing attention, since includes different types of scaffolds used for cell adhesion, proliferation, and differentiation [35,36]. Furthermore, different platforms of cell stimulation in vitro: compression, tension, fluid flow, hydrostatic pressure have been developed to study differentiation, proliferation, metabolism processes of cells or tissue explants [37,38]. In addition to mechanical stimuli, hypoxia is also known to be critical for chondrogenic differentiation in vitro, similarly to native cartilage: oxygen is supplied to chondrocytes by diffusion from the synovial fluid, with tension ranging from 1% O_2_ in the deep zones to 10% O_2_ at the surface [39,40].

Classical chondrogenic differentiation cocktail includes high glucose, serum-free medium, insulin-transferrin-selenium (ITS), dexamethasone, L-proline, ascorbic acid phosphate, and growth factors, most commonly transforming growth factor, beta 3 (TGF-β_3_). ITS promotes cell proliferation, formation of cartilage specific proteins and reduces dedifferentiation of mature chondrocytes [41]. Dexamethasone is a synthetic glucocorticoid, which promotes chondrogenic differentiation of MSCs by enhancing TGF-β_3_-mediated upregulation of collagen type II, as well as cartilage matrix-sulfated proteoglycans [42]. L-proline is necessary to stabilize the collagen α-helix conformation [43]. Therefore, the existence of L-proline in medium for chondrogenic differentiation contributes to collagen formation. Ascorbic acid phosphate is another bioactive supplement essential for chondrogenic differentiation in vitro, as it promotes cartilage ECM production [44]. Although each of these components is significant for a qualitative chondrogenic differentiation response, growth factors are among the most important factors to stimulate MSCs chondrogenic differentiation. TGF-β_3_ is a major growth factor used to induce chondrogenic differentiation in bone marrow derived MSCs, whereas bone morphogenetic proteins (BMPs), fibroblast growth factors (FGFs), and insulin growth factors (IGFs) are also commonly used as efficient stimulants of chondrogenic differentiation of different origin stem cells In Vitro [27,45].

### 3.2. Effects of Mechanical-Load on Chondrogenesis In Vitro

Mechanotransduction (mechano + transduction) is any of various mechanisms by which cells convert mechanical stimulus into electrochemical activity. In the cartilage, mechanical load is transduced to the chondrocytes were ensures their nutrition, stimulates ECM production and maintains chondroprotective and anti-inflammatory effects in the joints [46,47]. Under physiological conditions, compressive modulus of articular cartilage varies from 0.4–2.0 MPa [48]. Dynamic compression of cartilage results in matrix deformation, high pressure gradients, fluid flow, streaming potentials and currents, and physicochemical changes. Cartilage deformation leads to the chondrocyte deformation, which is important for signal transduction via membrane ion channels, cytoskeletal mechanotransduction or a range of other putative mechano-sensitive protein activation [49,50,51], which are known to modulate chondrocyte viability, gene expression, stimulate the synthesis of cartilage ECM proteins [37]. Furthermore, mechanical compression affects the intracellular Ca^2+^ concentration in chondrocytes. This process is regulated mainly by two ways: direct mechanical activation of Ca^2+^ -dependent channels and indirect change of membrane potential, which is maintained through voltage-operated calcium channels (VOCC). The most sensitive to mechanical impacts are calcium ion-conducting channels, such as L-type calcium channels that are particularly important for proper functional state of cartilage. However, the activation of L-type calcium channel functioning is associated with the pathogenesis of OA and VOCC are seen as the potential therapeutic targets ameliorating OA severity [52,53].

## 4. Techniques/Methods of Mechanical Loading In Vitro

Mechanical loading is an integral part of the environment of articular cartilage and crucial for its development and maintenance in vivo [54], therefore efforts have been made to introduce these forces into cartilage engineering as additional factors to choosing the right combination of cells, scaffolds, and bioactive materials [55]. However, as in vivo cartilage is affected by a variety of different forces, it is necessary to pinpoint the specific combinations of different mechanical stimuli and develop regimens that produce optimal chondrogenic effects [56]. Even though abundant studies are published about cartilage engineering, and specifically mechanical loading techniques, there is still no consensus on the specific characteristics and loading protocols of mechanical stimuli application that could be employed as standard cartilage engineering strategies In Vitro [57]. A range of mechanical stimulation systems have been produced seeking to reproduce the forces that the articular cartilage tissue endures in vivo and which are reported to affect chondrogenic differentiation.

**Figure 2:**
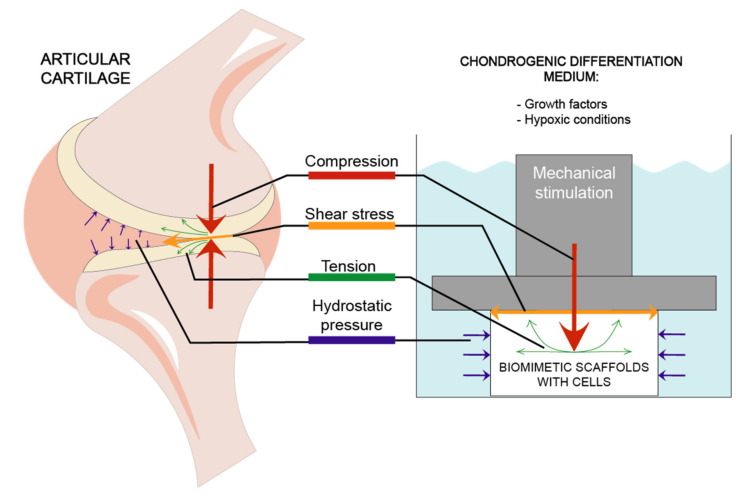
Different mechanical factors affecting articular cartilage and biomimetic scaffolds during chondrogenic differentiation.

Stimulation models most frequently used in studies are focused on 4 types of mechanical forces: compression, hydrostatic pressure, shear stress and tension [56,58] which are demonstrated in Figure 2.

The results of such models are highly dependent not only on those types of forces, but also on whether they are constant (i.e., static loading) or cyclically change throughout the experiment (i.e., dynamic loading), whether they are applied continuously or intermittently and whether or not they apply simultaneous loading of multiple types of forces [56]. For example, chondrocytes in cultures under dynamic stimulation benefited from increased nutrient accessibility resulting in higher viability as compared to static regiments [56].

Direct uniaxial static compression on a tissue surface is the most frequently studied mechanical stimulation strategy in cartilage tissue engineering [58]. Such bioreactor is simple to design, as it only requires basic weights placed on cartilage constructs [56]. However, static compression alone does not stimulate proteoglycan and protein synthesis as was shown with cyclical compression [59]. Therefore, in order to improve chondrogenesis and simulate physical conditions that are more similar to those exhibited in articular cartilage *in vivo*, dynamic compression bioreactors are applied with mechanisms such as pistons and tappets [58].

Dynamic mechanical loading has a positive effect on chondrogenic gene expression and biomechanical moduli [16]. So far, there is little standardization in cartilage tissue engineering, therefore different culture conditions, cell types, cultivation protocols, and bioactive molecules result in varying outcomes under similar mechanical loading conditions [16]. However, dynamic compression protocols involve frequencies around 1 Hz, mechanical loading amplitudes within the frame of 5–10%, daily compression periods of 1–4 h/day and loading durations of at least 7 days [57]. An important aspect of uniaxial compression is that the effect of loading is not imposed homogenously throughout the tissue, which results in depth-dependent variation. This leads to a heterogenous collagen deposition in MSC containing constructs which is highest on the surface [60].

Hydrostatic pressure—i.e., the application of uniform mechanical loading on all tissue surfaces—is also used in cartilage engineering. In comparison to uniaxial compression, bioreactor systems of hydrostatic pressure have even more varied schemes. Hydrostatic pressure values ranging from 0.1 MPa to 10 MPa and loading durations of 1–4 h/d have been demonstrated to enhance MSC chondrogenesis [61]. However, the optimal loading conditions for chondrogenesis remain unclear due to the differences between studies in parameters such as loading and interval duration, interval timing as well as frequency and peak pressure if dynamic loading regimes are applied [61].

Shear stress is the force that deforms an object by shifting its layers in relation to one another. As it affects the cartilage in vivo, the effect of shear stress was introduced to cartilage models, mostly alongside either fluid flow or compressive loading. The introduction of shear stress in some mechanical loading protocols resulted in upregulated expression of pro-chondrogenic genes and proteins [62]. Although not as frequently investigated as other forces, shear stress also causes specific benefits to MSC chondrogenesis, such as enhanced fiber organization and integration, that cannot be produced by compression or hydrostatic pressure alone [63].

Although relatively rarely used for chondrogenesis research, tension bioreactors have also been developed. Dynamic tension systems were reported to increase GAG synthesis in MSCs undergoing mechanical constriction [64]; however, in general, tension loading seems to rather favor osteogenic response, especially when compared to dynamic compression, which induced chondrogenesis [65].

Several issues should be noted regarding protocols of cartilage engineering utilizing mechanical loading. Most research groups use in-house bioreactors resulting in unreliable validation of the applied forces and limited method replicability, which makes comparison of data difficult and inconclusive [66]. Commercial mechanical stimulation devices for 3D tissue-engineered grafts are also currently available (some bioreactors are reviewed by Ravichandran et al. [67]), allowing comparisons between studies, however often their prices and maintenance are costly, which might be one of the factors encouraging research groups to develop their own biomechanical reactors [68]. Furthermore, mechanical loading bioreactors can only be used on certain culture models. For example, only 3D scaffolds and explants can be reasonably subjected to fluid flow, while tension is mostly analyzed on 2D-monolayer cultures [58]. The outcomes of mechanical loading also depend on the type of hydrogel used for scaffolding. Dynamic compression loading on MSCs was shown to upregulate synthesis of chondrogenic differentiation markers in moderately adhesive, as opposed to non-adhesive or highly adhesive hydrogels while under hydrostatic pressure, the induction of chondrogenic responses is most effective when the encapsulating hydrogel is highly adhesive [69].

## 5. Biomimetic Scaffolds for Mechanical Load-Based Chondrogenic Studies In Vitro

### 5.1. Synthetic Hydrogels

Hydrogels have been extensively used as matrices for drug delivery and as scaffolds for tissue engineering [70]. Hydrogels made of synthetic polymers have been widely used in tissue engineering applications. PEG-based hydrogels have been the most extensively investigated synthetic polymer for tissue engineering gelation in situ due to its excellent biocompatibility. The major problem of PEG hydrogels is its low degradability, which is common feature for other synthetic polymers. Thus, degradable crosslinkers need to be incorporated into synthetic hydrogels [71]. Nevertheless, non-degradable polymer debris still retains in the engineered tissue. On the other hand, natural polymers usually possess good biocompatibility and biological signaling. However, the mechanical strength of natural polymer hydrogels is usually insufficient to ensure the biomechanical environment *in vivo*. Adequate crosslinking is needed to increase the mechanical strength of natural polymer-based hydrogels.

The mechanical strength of synthetic hydrogels/scaffolds is often tested and expressed as Young’s modulus. Mechanical strength highly depends on porosity and stiffness of scaffold, which are regulated by a crosslinking mechanism. It is worth noting that porosity and structure of scaffold highly affect cell adhesion [72,73], therefore, specific crosslinking methods should meet biological and nutrient-transport needs, as well as requirements of mechanotransduction [74]. The increased pore size in scaffold might affect cell adhesion, on the other hand, the pore size must be spacious enough to allow cell migration. Moreover, it was shown that mechanical properties of scaffold also depend on the wetness level, which increase inferior mechanical strength [9]. Thus, in order to synthesize an efficient scaffold/hydrogel for cartilage tissue regeneration, a lot of questions should be answered regarding its components and crosslinking methods, which are the major points in designing mechanically stable, biocompatible, and biodegradable constructs.

### 5.2. Physical and Chemical Crosslinking Methods

Application of gelling tissue engineering systems in situ has attracted a lot of attention due to the ease of their administration during surgical procedures. The mechanisms for in situ gelling are classified as physical and/or chemical crosslinking. The means of physical crosslinking including ionic interaction, sol–gel transition and substrate–ligand binding employs reversible physical interactions between polymer chains and matrix network [75]. Physical crosslinking does not involve chemical reactions, so it is less toxic in comparison to chemical crosslinking. However, physically crosslinked hydrogels usually possess insufficient mechanical strength to resist mechanical stress in the body [76]. The methods of chemical crosslinking include: polymerization, Michael addition reaction, photo-initiated and enzyme-mediated crosslinking [75]. Chemical crosslinking is a more versatile method for the fabrication of mechanically stabile hydrogels with controlled degradability. However, cytotoxic species generated during the crosslinking process in situ raise concerns of undesirable reactions with bioactive molecules and cells.

In general, the biggest advantage of crosslinking reactions of natural or synthetic polymers in chondrogenesis studies is their ability to control shapes and sizes of scaffolds and consistency of injectable substance carrying drugs or active compounds. The crosslinking of polymers allows to encapsulate bioactive compounds with controlled release properties. Crosslinking also allows regulation of mechanical strength, mechanotransduction, modulus of elasticity, cell adhesiveness, porosity, degradation rate, and mode of used scaffolds and hydrogels that extend their application in chondrogenesis.

## 6. ECM and Other Natural Component-Based Scaffolds for Chondrogenesis Studies under Mechanical Load

**Figure 3:**
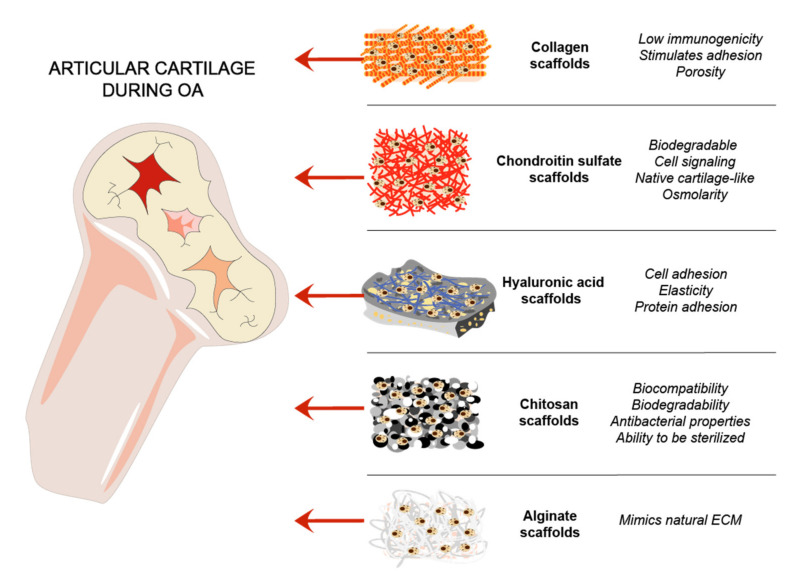
ECM and other natural components-based scaffolds for cartilage regeneration. Steps of cartilage regeneration applying cells incorporated into biomimetic scaffolds.

Cartilage natural composites were always of interest for cartilage tissue regeneration, due to mimicking the natural environment in vivo [36,77]. Collagens are routinely used as a biomimetic tool for cartilage regeneration. GAGs are linear chains of negatively charged polysaccharides that are divided into sulfated GAGs such as CS, heparin, KS, and non-sulfated GAGs represented by hyaluronan [78]. HA is an important joint component, which acts as a lubricant and maintains mechanical properties of a joint [79]. Chitosan and alginate are natural polymers that were used for cartilage scaffold development and showed promising results [36] (Figure 3).

However, there is a lack of chondrogenesis studies in scaffolds under mechanical loading. Therefore, we gathered most relevant studies that used scaffolds and mechanical loading for chondrogenic differentiation (Table 1).

### 6.1. Collagen-Based Scaffolds

Collagen (250–300 kDa) is the main structural component of cartilage, which provides a strong physical support and maintains structural cartilage integrity. Collagen scaffolds are most widely used in cartilage tissue engineering, as they offer perfect biocompatibility, porosity, low immunogenicity and stimulate cell adhesion, migration, and differentiation [95]. However, these constructs have poor mechanical properties, which limits their use in most load bearing studies, where mechanical stability of a construct is a crucial factor for developing clinically relevant cell-based 3D structures. It has been shown that Young’s modulus pure collagen scaffolds is around 6.1 ± 0.3 GPa [96,97]. Therefore, collagen-based scaffolds are often modified with a number of different natural/synthetic polymers, using chemical or physical crosslinking methods, which would tune up mechanical stability in both, in vivo and In Vitro systems.

Collagen type I and type II scaffolds usually have been fabricated in three forms: hydrogels, porous sponges, and nanofibers [7]. Hydrogels are easily injectable, forming a branch of three-dimensional collagen network and exhibiting good cell and tissue biocompatibility properties [98,99]. Porous sponges consist of membrane-like wall, which allows the cells to evenly distribute [73,100], while nanofibers consist of a network of collagen fibers, mimicking structural cartilage fibers in vivo [101]. Mechanical compression was less applied on seeded on stem cell loaded collagen scaffolds however, several studies suggest its beneficial effects. Equine collagen type I scaffolds with human bone marrow MSC (BMMSCs) were stimulated with 1% amplitude sinusoidal strain at 0.01, 0.1, 1 Hz for 10 cycles each, which resulted in an improved chondrogenic phenotype of the construct compared to not compressed ones [81]. Other study also showed that collagen I scaffolds with BMMSCs upregulated cartilage-specific genes SOX9, aggrecan, collagen II expression after compression with 10% peak compressive sinusoidal strain at 1 Hz frequency for 2 h/day [80].

Moreover, collagen-based scaffolds are very popular and have gained great achievements not only in cartilage engineering field, but also in skin, bone, tendon, ligament, and blood vessel studies [95]. However, much work should be done in order to find the best solution for an efficient treatment of cartilage applying natural or synthetic scaffolds.

### 6.2. Chondroitin Sulfate and Its Derivative-Based Scaffolds 

Chondroitin sulfate (CS) (average molecular weight of 15 kDa) is a sulfated GAG, which is a primary component of cartilage ECM and plays an important role in cartilage tissue functioning. When incorporated in a hydrogel, CS introduces negative charges, which elevate the local osmolarity similar to native cartilage. Also, naturally-derived CS scaffolds are attractive for biological application due to their positive cell signaling, cell-interactive and biodegradable properties. The main problem of low mechanical stability and short-term duration of CS scaffolds in vivo is solved by combining them with synthetic components such as PEG or polyacrylamide (PAM) [102].

CS-based scaffolds have been shown to positively affect chondrogenic differentiation [103,104]. A study made in 2019 has described thiolate chondroitin sulfate scaffolds combined with poly-ethylene glycol as an efficient cartilage mimetic-hydrogel that together with dynamic loading stimulated chondrogenic response in induced-pluripotent stem cells, with some hypertrophy limitations [105]. Therefore, CS is usually mixed with other different components, like gelatine or synthetic PEG, dynamic loading scaffolds in order to reach better chondrogenic differentiation result. For example, dynamic loading from 0% to 15% amplitude strain in a sinusoidal waveform at frequency of 0.3 Hz has downregulated hypertrophic proteins Col X, RUNX2, and collagen I in MSCs [106]. Moreover, different types of dynamic loading (5% strain 0.3 Hz (1.5% s^−1^); 10% strains 0.3 Hz (3% s^−1^); 5% strain 1 Hz (5% s^−1^); 10% strain 1 Hz (10% s^−1^)) was shown to upregulate collagen II gene expression [84].

### 6.3. Hyaluronic Acid-Based Scaffolds

Hyaluronic acid (HA), also called hyaluronan, is an anionic, nonsulfated glycosaminoglycan with size range from 0.4 to 20,000 kDa, naturally occurring polymer, distributed mostly in connective epithelial and neural tissues. The molecular weight of HA strongly depends on the source and can have different affects, i.e. 0.4–4 kDa HA acts as heat shock inducer, 6–20 kDa—has immunostimulatory and angiogenic properties, 20–200 kDa participates in embryonic development and wound healing, while >500 kDa can function as space filler and natural immune suppressant [107].

HA is also found in viscose body fluids, such as synovia, which pays an essential role in reduction of friction during movement between the articular cartilages of synovial joints. Increased dilution of HA in synovial fluid results in lower cartilage elasticity, since viscoelastic properties of HA reduce transmission of mechanical force to the cartilage [108]. The impaired balance between mechanical stress and protective components in the joint leads to the quicker wearing out resulting in OA [3]. In addition, HA as a main component of GAGs, interacts with the cell trough CD44, a HA receptor, and stimulates chondrogenic differentiation. CD44 can also interact with other ligands, such as osteopontin, collagens, and matrix metalloproteinases and promote chondrogenic gene expression [109].

Early studies have shown that including viscoelastic compounds into the osteoarthritic joint improved its function [110]. Viscosupplementation (VS) with different HA preparations (low and high molecular weight HA), can be considered when pain relief therapy cannot be used and patient is intolerant to analgesics or NSAIDs. HA might act as a viscous fluid or more elastic solid depending on the strengths of the shear stress and dissolved oxygen—i.e., both *S. zooepidemicus* G1 growth and HA synthesis were slower under anaerobic (below 30% of dissolved oxygen) conditions and the HA molecular mass was only 1.22 ± 0.02) × 10^6^ Da, whereas high level of dissolved oxygen (above 50% of dissolved oxygen) favored the increase of HA molecular mass, which reached a maximum value of 2.19 ± 0.05 × 10^6^ Da. Similar to the low oxygen level, a high shear (600 rpm) stress delayed the rate of HA synthesis and decreased the HA molecular weight compared to the low shear stress (150 rpm) [111]. Seems that a low concentration of dissolved oxygen and high shear stress (conditions similar to the cartilage) prevents HA polymerization. It was also shown that direct injections of HA in the joint space is useful in the treatment of various OA joints, especially knee in elderly [112,113]. Treatment of OA shoulder, carpo-metacarpal, hip, and ankle in elderly were encouraging but inconclusive. However, the vs. of HA directly to the osteoarthritic hip is not recommended since there was scarce evidence of its efficacy up to 3 months and no efficacy at 6 months [114,115].

Since HA is a viscous solution used mostly for intraarticular injections, the main scientific goal is to improve mechanical properties of HA. Therefore, to form 3D HA hydrogels, scientists are using various types of synthetic cross-linking agents and/or polymerization techniques.

### 6.4. Mixed Type HA Hydrogels for Chondrogenesis under Mechanical Load

#### 6.4.1. Collagen and Hyaluronic Acid Hydrogels

Collagens are used in combinations with HA and have shown strongly improved chondrogenic differentiation of adipose-derived mesenchymal stem cells (hAMSCs) [116]. Collagen type I and HA hydrogels (Col-HA) were fabricated by direct mixing different amounts of HA (0–5%) into collagen type I solution before gelation. The chondrogenic differentiation increased in Col-HA gels compared to just Col measured by oxygen consumption and gene expression. However, the effect of HA strongly depended on its concentration—the change of HA concentration from 0.5 to 5% effectively changed the Young’s modulus from 5.8 to 9.0 kPa. The highest HA concentration (5%) used in Col-HA gels showed best chondrogenic differentiation results [116].

#### 6.4.2. Fibrin and Hyaluronic Acid Hydrogels

Fibrinogen (approximate molecular weight of 340 kDa) is glycoprotein normally present in human blood plasma. Fibrinogen, by the action of serine protease thrombin, forms insoluble or gel form monomer fibrin clots that are important to prevent blood loss [117]. Fibrinogen monomer fibrin, in combination with HA, is most common material used for tissue regeneration purposes since HA is able to directly interact with fibrin precursor fibrinogen and make reversible ionic interactions. In addition, fibrin contains native arginine-glycine-aspartic acid sites for cell attachment, that are absent in HA hydrogels. Fibrin was tested in stimulation of chondrogenic differentiation under compressive force using three different frequencies (0.1, 0.5, and 1.0 Hz) and human BMMSC proliferation, viability and differentiation have been investigated. It has been demonstrated that fibrin constructs supported MSCs chondrogenesis under cyclic compression [118]. Even though, the fibrin by itself can be used to promote chondrogenesis, though HA hydrogel showed better mechanical strength than fibrin. Thus, combining of HA and fibrin resulted in superior hydrogel, which mixed with BMMSCs and injected directly into joint repaired OA articular cartilage [85]. In addition, ultrasound stimulation of HA-fibrin hydrogels improved chondrogenic differentiation of BMMSCs [87].

Study done with low-intensity ultrasound (LIUS) also showed, that mechanical stimulation of fibrin-HA and rabbit MSC (1.0 MHz and 200 mW/cm^2^) for four weeks’ enhanced production of GAGs and collagen. The combined fibrin-HA hydrogels under higher mechanical strength were more effective than alginate-HA. The best compressive strength for fibrin-HA and fibrin-HA with LIUS samples measured at four weeks were: fibrin-HA around 11 MPa/%, fibrin-HA with LIUS around 14 MPa/% [87].

#### 6.4.3. Alginic and Hyaluronic Acids Hydrogels

Alginic acid, also called algin or sodium alginate (Alg), is a 76–190 kDa polysaccharide widely distributed in the cell walls of brown algae, which is hydrophilic and forms a viscous gum when hydrated. Alginic acid is composed of α-L-glucuronic acid (G) and β-D-mannuronic acid (M) and can be obtained from brown seaweed and pathogenic bacteria such as Pseudomonas aeruginosa [119]. The way in which M and G units are arranged in the alginate chain, the overall ratio of the two units (M/G) in a chain can vary from one species of seaweed to another. Generally, alginates with a higher content of G will give a stronger gel; such alginates are said to have a low M/G ratio [120]. Alginic acid, after ionic cross-linking with metals such as sodium and calcium, compose salts known as alginates. Alginate hydrogels are widely used in regenerative medicine for different tissue engineering purposes, especially for bone and cartilage, since its structure is similar to glycosaminoglycan mimicking natural cartilage ECM.

Both, ionic and covalent, cross-linking methods have been investigated to generate hydrogels with broad mechanical properties. The enzymatically (in the presence of horse radish peroxidase (HRP) and hydrogen peroxide) modified tyramine, sodium alginate, and sodium hyaluronate was successfully used to improve metabolic properties of murine chondrocyte cell line ATDC-5 [121]. The 1% of sodium alginate have an elastic modulus of 11 kPa, whereas changing alginate and calcium concentrations, and using ionic crosslinking it was possible to variate elastic modulus from 1 to 10 kPa [121,122]. Photocrosslinking methacrylate and alginate using photoinitiator allowed to make 3D chondrocyte constructs with aggregate moduli ranging from 10 to 20 kPa [123].

Both alginate and HA have been very popular as biomaterials for hydrogels; however, both are lacking some necessary properties—alginate does not interact with the cells and proteins, whereas HA does, it is why alginate requires cross-linking agents to produce stiffer hydrogel, which might have some cytotoxic side effects. Hydrogels of alginate modified with low molecular weight hyaluronate tend to display better mechanical stiffness and chondrogenesis, provide suitable porosity network, make a positive impact on transportation of nutrients and distribution of newly synthesized cartilage matrix [124]. It was shown that addition of HA to alginate enhanced chondrogenesis of mouse chondrocytes in comparison to alginate only gels and chondrogenic differentiation depended on amount of HA, not alginate [124]. As the weight ratio of hyaluronate to alginate increased from 0.1 to 1, the gel stiffness (G′) changed significantly from 3.2 ± 0. 7 kPa to 9.1 ± 0.7 kPa. The best effect on Sox9 and aggrecan expression was observed at ratio of hyaluronate to alginate 0.5 and 1 [124]. The co-encapsulation of TGF-β_3_ containing alginate microspheres with human BMMSCs and HA hydrogels have been also used to develop implantable constructs for cartilage repair [125]. The intra-articular injection of alginate-chitosan beads in experimental osteoarthritis lesions in rabbit also decreased OA cartilage lesions without inflammatory signs [126].

#### 6.4.4. Chitosan and Hyaluronic Acids Hydrogels

Chitosan is a positively charged linear 5–200 kDa polysaccharide, commonly found in exoskeleton of crustacean shellfishes and insects, shrimp waste, crab and lobster, and cell walls of fungi. Chitosan is N-deacetylated derivative of chitin obtained by treatment of chitinous material with highly concentrated potassium hydroxide under boiling temperature or naturally in seaweed and mushroom by chitin deacetylases immediately after the biosynthesis of chitin [127].

However, it was shown that artificial 10% removal of N-acetyl groups from two different (α and β) chitin nanofibers cannot produce chitosan; the deacetylation should be not less than 50%) [128]. Partial deacetylation of chitin makes chitosan, unlike chitin, water soluble in a week acid media and extend its application in medicine [129]. Chitosan has also attracted attention due to its biocompatibility, biodegradability, antibacterial properties, ability to be sterilized, therefore, be used in tissue engineering as cells carrier and deliverer to the tissue [36,130,131]. In addition, chitosan has similar structure as glycosaminoglycans and it is believed to be able to induce or support chondrogenesis [132]. Scaffolds made from chitosan can be used in various forms—such as gels, films, fibers or sponges [11]. Despite of advantages, chitosan scaffolds also have few disadvantages, such as low water solubility, poor mechanical strength, and stability. To eliminate mentioned drawbacks, chitosan, can be combined with other natural components such as cartilage ECM composite, agarose, alginate, and other [133,134,135].

The chitosan-HA-based biomimetic matrix in conjunction with adipose-derived MSC cells better supported formation of articular hyaline cartilage than standard chitosan-based construct. However, the chitosan-HA-based biomimetic matrix needed a cocktail of morphogens such as TGF-β_3_, BMP6, dexamethasone, L-ascorbic acid-2-phosphate, L-proline and ITS+1 for chondrogenic differentiation [136]. The chitosan has been also successfully used for coating of synthetic scaffolds such as poly L-lactide-co-ɛ-caprolactone scaffolds in chondrogenic differentiation, while its application in mechanotransduction studies so far is limited [137].

#### 6.4.5. Other Types of Combined Hydrogels

An injectable and biodegradable hydrogel system comprising HA and tyramine (HA-Tyr) conjugates can safely undergo covalent cross-linking in vivo after the addition of small amounts of peroxidase and hydrogen peroxide [138]. Hydrogels crosslinked by the HRP conjugation with tyramine have also been prepared from alginate, dextran and carboxymethylcelullose [139]. HA-Tyr hydrogels were shown to meet the basic chemical, physical, and technical requirements required for cartilage regenerating hydrogels, i.e., HA-Tyr hydrogels have compressive strength of 5–11 kPa and have been applied as biomimetic matrices for caprine MSCs in cartilage tissue engineering [140].

Methacrylated hyaluronic acid (MeHA) hydrogels with photo-encapsulated MSCs were also used in cartilage tissue engineering. MeHA hydrogels seeded with MSCs preconditioned by a chondrogenic induction medium and stimulated for 14 days by dynamic compression (10% peak compressive sinusoidal strain at 1 Hz frequency, superimposed on a 5% compressive tare strain) exhibited better mechanical characteristics, such as higher Young’s modulus and maximum load, as compared to mechanically unstimulated controls [141]. In addition, following implantation into osteochondral defects in rats, the compressed hydrogels were shown to produce higher quality surface tissue at the defect area [141]. However, the mechanical test performed on the constructs treated with dynamic preloading increased the Young’s modulus by more than 200% (from ~20 kPa to ~60 kPa) compared to the constructs treated only with free loading (regular chondrogenic media) counterparts. Study also suggest that dynamic compressive loading may increase nutrient transport into scaffolds, thus enhancing production and distribution of MSCs-produced cartilage matrix [141]. Nanofiber-HA membrane system made from the negatively charged high molecular weight HA solution and its contacts with positively charged peptide amphiphile (PA) molecules, also showed better result in treatment of early and late rat model OA stages compared to commercially available hyaluronic acid supplement Hyalgan^®^ and Synvisc [142]. Moreover, using cross-linked methacrylate and HA scaffolds, high concentration of MSC (up to 60 × 10^6^ cells/mL) and mechanical stimulation (0.02 N for 5 min to 1000 sec relax and 1% sinusoidal deformation was applied at 1.0 Hz for 63 days) highly improved chondrogenic differentiation [88]. HA hydrogels formed via photocrosslinking also provide stable 3D hydrogel environments that support the chondrogenesis of mesenchymal stem cells [143].

## 7. Discussion and Future Directions

Articular cartilage is an avascular tissue with low self-healing capability. ECM of articular cartilage together with chondrocytes are highly organized structure withstanding mechanical load. However, if lesions occur, host chondrocytes have very limited capacity to rebuild the cartilage tissue and replace the dead cells. Thus, surgical and/or clinical interventions, including partial or total joint replacement are often necessary [7]. Therefore, cartilage tissue engineering using different kinds of implantable scaffolds is of high clinical interest. In this review we have aimed to discuss natural, biodegradable, mainly cartilage ECM-based scaffolds—such as collagens, chondroitin sulfate, hyaluronic acid, chitosan, fibrin, and alginate—and their application for chondrogenic differentiation under mechanical load. Data presented in Table 1 show promising results on chondrogenic differentiation of stem cells cultivated on ECM-based matrices under different intensity of mechanical loads, resulting in beneficial mechanotransductive effects. Among abundant studies using natural scaffolds or hydrogels for chondrogenic differentiation purposes, the studies with mechanical load are rather scarce. Collagen fibrils are key players in multiple human connective tissues, including interstitial matrix, basement membranes, bone, and definitely cartilage matrix. The formation of collagen fibrils depends largely on individual collagen molecules and amino acids that compose each helix in tropocollagen. The individual collagen fibrils are naturally flexible, which is supported by other ECM components. Therefore, adequate mechanical compression resistance properties are originally characteristic to cartilage ECM structures. To mimic the mechanical stability of natural cartilage In Vitro is one of the main scientific goals.

It has been shown that MSCs efficiently produce cartilage ECM under chosen mechanical load protocol: 1–10% of sinusoidal strain with 1Hz frequency of compressive force is being applied for at least 2 h/day [80,81,82]. Similar compressive forces were also shown to have a positive effect on chondrogenic differentiation of MSCs embedded in chondroitin sulfate scaffolds, which are frequently used in combination with other synthetic polymers to ensure stiffness of the constructs [83,84]. Due to its specific binding to the cells through CD44 receptor, HA is also very popular for cartilage regenerating purposes. Therefore, HA is most often used in orthopedic surgery for direct injections into damaged joints, though it is mechanically unstable component. To increase its stability, HA is usually mixed with other compounds, such as collagen, fibrin, alginate, chitosan and others. Mixed hydrogels were shown to significantly improve chondrogenic differentiation of cells in vitro, with or without different mechanical compression protocols, as compared to single components of the hydrogel [85,86,87,88].

So far there is no unique scaffold and/or cell combination suitable for all types of cartilage repair, but improvement of tissue engineering techniques In Vitro is obvious and shows promising results for their therapeutic application *in vivo*. In addition, the scaffold/hydrogel material for chondrogenic regeneration strongly depends on the used cells, chosen active compounds (differentiation protocol) and other differentiation conditions to be used. Studies in this field are still on the way of new discoveries, however according to the current studies—compression force of 1 Hz frequency, 10–15% of sinusoidal strain for at least 30 min a day is a basic criterion to significantly upregulate chondrogenic genes in the cells after 7 days of culturing (see Table 1). These experimental findings In Vitro, are in agreement that regular moderate walk or other exercises are beneficial for human joints.

In conclusion, natural component-based scaffolds/hydrogels are of great interest in chondrogenic differentiation of MSCs, chondrocytes or pluripotent stem cells due to their biodegradability, biocompatibility and regulated mechanical strength. By adopting contemporary chemical technologies and/or compounds, the natural material-based constructs can withstand excessive mechanical load while transducing relevant mechanical signals to the cells, which is important for qualitative chondrogenic regeneration studies and cartilage tissue engineering In Vitro. Studies in this direction are expanding and hold a firm basis for future experimental studies in vivo and translation to clinical applications.

## Figures and Tables

**Table 1:** Natural compound scaffolds/hydrogels used in chondrogenic differentiation under mechanical compression.

Scaffold Base	Additional Compounds	Cell Type	Compression Parameters	Compression Duration	Results after Compression	Reference
**Collagen**	Collagen I	Human MSCs	10% peak compressive sinusoidal strain at 1 Hz frequency	2 h/day, 1, 4, 7, 14 and 21 days	Upregulated cartilage specific genes: AGG, COL2A1 and SOX9, and prevented expression of COL10A1, and COL1A2.	[80]
	Collagen I	Human MSCs	1% amplitude sinusoidal strain at the three frequencies of 0.01, 0.1, 1 Hz for 10 cycles at each frequency	-	Resistance to compression, increased dynamic properties of the scaffold, more marked viscoelastic behavior over time	[81]
	Alginate	Human MSCs	10% or 15% cyclic compressive strain	4 out of 24 h for up to 21 days	Increased expression of CBFA-1, Sox9, and aggrecan under 15% cyclic compressive strain alone	[82]
**Chondroitin sulfate**	PEG	Human MSCs	Dynamic loading from 0% to 15% amplitude strain in a sinusoidal waveform at a frequency of 0.3 Hz	0.5 h on, 1.5 off, repeated for 16 h followed by 8 h off, during 1 week	Downregulated Col X, RUNX2 and collagen I/II protein expression	[83]
	PEG	Human MSCs	Dynamic loading 5% strain 0.3 Hz (1.5% s^−1^); 10% strains 0.3 Hz (3% s^−1^); 5% strain 1 Hz (5% s^−1^); 10% strain 1 Hz (10% s^−1^)).	1 h/day for a week	Upregulated collagen II gene expression	[84]
**Hyaluronic acid**	Fibrin	Human MSCs	Compressive modulus at 20% strain, 3.39 ± 0.91 kPa to 6.76 ± 0.52 kPa	-	Eased collagen type 1 expression and an increase Sox9 expression	[85]
	Hyaluronan- gelatin composites	Human BMMSCs		4 h/day in the first 7 days of culture	Proteoglycan and collagen contents were significantly higher in the loaded samples compared to unloaded controls	[86]
	Fibrin	Rabbit BMMSCs	Mechanical compression of low-intensity ultrasound (LIUS)	4 weeks of LIUS (1.0 MHz and 200 mW/cm^2^)	LIUS induces chondrogenic differentiation of MSCs without TGF-ß3 treatment and increased chondrogenic markers	[87]
	Methacrylate	Calve BMMSCs	0.02 N for 5 min to 1000 sec relax and 1% sinusoidal deformation at 1.0 Hz	3, 7, 21, 63 days	Increase in GAG production after 63 days under mechanical load	[88]
**Chitosan**	Collagen	Rabbit chondrocytes	Cyclic compression of 40% stain, 0.1 Hz	30 min/day for two weeks	Enhanced cells proliferation and GAGs deposition. 82% degree of chitosan deacetylation	[89]
	Oxidized dextran, and teleostean	Human MSCs	0.02 N preload for 500 sec, this consisted of 20% stain, 2 Hz, 0.12 and 0.96 MPa	42 days	Upregulation of aggrecan and collagen II mRNA, and increased GAG and collagen content. 85% degree of chitosan deacetylation	[90]
	Agarose	Human MSCs	Dynamic compression loading was applied at 1 Hz	1 h/day, 14 days.	Total GAG content and GAG/DNA content was significantly higher, as compared to unloaded control.	[91]
	Silk fibrin and nano- hydroxyapatite	Rat BMMSCs	10% compressive strain, 0.5 Hz	2 h action + 4 h pause/cycle, 4 cycles/day	Upregulation of chondrogenesis markers (Aggrecan, Sox-9, and collagen II. 92( ± 2.1)% degree of chitosan deacetylation	[92]
**Alginate**	-	Rabbit adipose-derived MSCs	1 Hz, with air pressure 21 kPa and different stain magnitudes (0–20%).	2 h per day, 6 days a week	Expression of Col II, SOX9 and aggrecan w/o TGF-ß	[93]
	-	Human MSCs	0.5 Hz frequency and 6813 ± 2195 Pa	21 d.	SOX9 and aggrecan gene expression of uncompressed group was low and increased 15 fold and 7 fold, respectively, for compressed group	[94]
